# Does exercise adherence during the COVID-19 pandemic contribute to improved subjective well-being? A cross-sectional study

**DOI:** 10.3389/fpsyg.2024.1448827

**Published:** 2024-07-31

**Authors:** Yonghuan Chen, Ning Fang, Yulong Zhu, Zhenyu Li, Qiuhan Zhu

**Affiliations:** ^1^School of Physical Education, Zhengzhou University, Zhengzhou, China; ^2^Department of Marine Sports, Pukyong National University, Busan, Republic of Korea

**Keywords:** COVID-19 pandemic, exercise adherence, positive mental character, subjective well-being, indirect effect

## Abstract

**Introduction:**

This study aims to investigate whether exercise adherence and positive mental character significantly affect subjective well-being among Chinese college students during the COVID-19 pandemic and whether positive mental character plays a mediating role.

**Methods:**

The study employed questionnaires, including the Exercise Adherence Scale, the Positive Mental Character Scale, and the Subjective Well-Being Scale, which were administered to students across seven universities in Henan Province, China. A total of 1,001 participants were analyzed in the final sample. Data were analyzed using SPSS 21.0 for descriptive statistics, independent samples T-test, correlation, and regression analyses. Furthermore, structural equation model with AMOS was conducted to examine the potential mediating effect of positive mental characteristics on the relationship between exercise adherence and subjective well-being.

**Results:**

The results indicated significant differences in exercise adherence, positive mental character, and subjective well-being between male and female participants, with males scoring higher in all three domains. Among Chinese university students during the COVID-19 pandemic, there was a significant correlation among exercise adherence, positive mental character, and subjective well-being. Exercise adherence was found to have a significant and positive impact on both positive mental character and subjective well-being. Additionally, it was found that positive mental character had a significant positive association with subjective well-being. The mediating role of positive mental character in the relationship between exercise adherence and subjective well-being was partially supported.

**Discussion:**

Exercise adherence among Chinese college students had a significant positive association on both positive mental character and subjective well-being throughout the COVID-19 pandemic. The exercise adherence can directly or indirectly enhance subjective well-being through its association with positive mental character. Therefore, positive mental and subjective well-being can be enhanced by consistent physical activity even during a pandemic.

## 1 Introduction

Adolescent college students are considered to be physically, mentally and emotionally more sensitive (Fuentealba-Urra et al., 2022). Research indicates that college students frequently encounter negative emotions, including boredom, anxiety, and frustration (Ramón-Arbués et al., 2020; Wang et al., 2024), and are at a notably elevated risk for depression compared to the broader population (Ibrahim et al., 2013). The advent of the COVID-19 pandemic has further exacerbated the situation, with the college student population witnessing a marked increase in psychological health issues compared to pre-pandemic levels (Volken et al., 2021). Consequently, addressing the mitigation of negative emotions and the enhancement of subjective well-being among individuals during the COVID-19 pandemic has emerged as a pivotal research focus for scholars. Subjective well-being is shaped by a multitude of factors originating from personal, educational, and familial contexts, with personality traits being recognized as the most consistent and potent predictors of this construct (Anglim et al., 2020). Positive mental character constitutes the affirmative aspect of personality traits and is a relatively stable psychological attribute that emerges from the interplay between an individual’s innate qualities and their acquired environment (Wang, 2010). This psychological attribute exerts a positive influence on, and can even be decisive in shaping, an individual’s thoughts, emotions, and behaviors, thereby establishing a foundation for their subjective well-being.

Physical activity has been demonstrated to effectively enhance an individual’s positive mental character and subjective well-being (Mansfield et al., 2018; Mahindru et al., 2023). Research in exercise psychology, informed by positive psychology, suggests that physical exercise contributes to the promotion of physical and mental health, as well as the cultivation of a robust physical and mental state. Such benefits extend to cognitive (Bidzan-Bluma and Lipowska, 2018) and personality (Piepiora et al., 2021) development, thereby significantly enriching an individual’s positive mental character and subjective well-being. Contemporary exercise psychology increasingly highlights the positive psychological outcomes and the fostering of positive mental character that accompany physical activity. Physical exercise is instrumental in eliciting emotions such as “flow experiences,” energetic states, and pleasant moods, while also reducing the likelihood of triggering anxiety and fatigue (Liu and Qi, 2016). This facilitates the emergence of positive psychological states and suppresses the onset of negative psychological tendencies (Zhang and Zhou, 2013). However, the extent to which exercise adherence continues to directly or indirectly associated with subjective well-being through positive mental character during the COVID-19 pandemic remains unclear. In addition, in the available studies, researchers have explored the relationship between the three variables in the context of the era of normalization. However, there is still a lack of studies that discuss this in the context of a global pandemic. People also suffer great psychological stress in the face of the pandemic’s threat to their health and lives. Therefore, this study aims to know the association between the three in the context of a global pandemic. This will help us to identify an effective intervention for promoting people’s mental health and enhancing subjective well-being when we encounter similar situations again in the future.

## 2 Theory and hypothesis

### 2.1 Theoretical background

The Health Belief Model (HBM) is a widely used theoretical framework in health behavior research. The HBM suggests that an individual’s health-related behaviors are influenced by their perceptions of the threat posed by a health problem and the benefits and barriers associated with taking action to address it (Green and Murphy, 2014). The HBM consists of several key constructs, including perceived susceptibility, perceived severity, perceived benefits, perceived barriers, cues to action, and self-efficacy (Green and Murphy, 2014). In the context of the COVID-19 pandemic, people have greater psychological pressure from viruses that cause harm to them. As a result, they are more likely to go and release stress by taking relevant measures, and physical exercise has not only proved to be an effective way of relieving psychological stress, but also an effective measure to promote the generation of positive psychology and enhance subjective well-being (Mahindru et al., 2023).

Broaden-and-Build Theory of Positive Emotions proposed by Fredrickson (2001) systematically explains the mechanisms by which positive emotions enhance mental health. The theory suggests that expansive thought patterns triggered by positive emotions can provide indirect and long-term adaptive benefits, which will help build lasting personal resources, including physical, psychological, intellectual, and social resources, which can help individuals cope with current or future threats. This in part predicts that positive mental quality as a positive emotion may be potentially linked to subjective well-being, and that this link persists even under the threat of a global pandemic.

### 2.2 The relationship between exercise adherence and subjective well-being

Exercise adherence is defined as an individual’s commitment to long-term, regular engagement in physical activity, with the antonym being exercise dropout (Chen, 2007). The positive emotion expansion theory related to adolescent physical activity posits that individuals can derive positive emotional experiences through exercise, which in turn enhances their motor cognitive abilities and broadens their operational framework for motor behaviors in sports contexts (Fredrickson, 2004). Furthermore, these positive emotional experiences are crucial in building enduring physical, cognitive, psychological, and social resources (Guo, 2015). Research across biological and psychological disciplines has consistently demonstrated the unique role of physical activity in emotion regulation, affirming its status as one of the most effective strategies for emotional management (Ji et al., 2010). Interventions employing various exercise programs have yielded significant improvements in subjective well-being, particularly among older adults who engaged in a 12-week tai chi regimen (Wang et al., 2023). Studies have also revealed a robust positive correlation between exercise adherence, self-efficacy, and subjective well-being among adults involved in Latin dance, where self-efficacy mediates the relationship between exercise adherence and subjective well-being (Tang, 2019). Additionally, a clear positive association exists between the intensity of physical activity and subjective well-being (Panza et al., 2019). Research indicates that both moderate- and vigorous-intensity exercises are linked to increased subjective well-being and decreased negative emotions among college students (Zhang et al., 2022). Ye and Guo (2023) surveyed 894 college students, the exercise adherence was found to be positively correlated with subjective well-being. Physical activity, peer relationships, and subjective well-being also showed significant correlations among college students (Chen and Yu, 2015). This suggests that such exercise intensities may be particularly beneficial for enhancing the well-being experiences of college students. A cross-sectional analysis involving 1,046 older adults during the COVID-19 pandemic further supports the notion that even light physical activity can alleviate the negative psychological impacts associated with social isolation and adherence to social distancing measures (Callow et al., 2020).

In summary, the available evidence supports a positive correlation between exercise adherence and subjective well-being, with even low-intensity physical activities being instrumental in fostering psychological well-being throughout the COVID-19 pandemic. Nonetheless, the literature lacks definitive findings on the specific impact of exercise adherence on the subjective well-being of college students amidst the pandemic context. Consequently, this study posits the following research hypothesis:

*H1*: During the COVID-19 pandemic, college students’ exercise adherence are significantly and positively associated with subjective well-being.

### 2.3 The relationship between positive mental character and subjective well-being

Positive mental character constitutes a subset of overall mental character, encompassing positive qualities such as self-confidence, optimism, and resilience, which emerge from the interplay between an individual’s innate potential and environmental influences (Lopez et al., 2006). These characters are distinguished by their stability, potential for growth, adaptability, and creative capacity (Li et al., 2017). Numerous scholars posit that positive mental character encompasses several key psychological attributes: it fosters enhanced work or academic performance; elevates individuals’ subjective well-being; and serves as a protective factor against mental illness, thereby contributing to the advancement of mental health (Tian, 2011). Positive psychologist Seligman and Csikszentmihalyi (2000) posits that individuals possess character strengths, which are unique positive qualities such as optimism, curiosity, and self-control. The authors argues that by fully leveraging these strengths in their daily lives, individuals can cultivate a profound sense of fulfillment and happiness. These strengths, comprising 24 distinct character strengths, form the foundation of well-being (Zhang, 2005). By honing these traits, individuals are better equipped to navigate life’s challenges and enhance their overall well-being (Linley and Joseph, 2004). Prior studies have corroborated the efficacy of these strengths in foretelling well-being and suggest that their strategic application can elevate an individual’s sense of well-being (Park et al., 2004). Notably, children and adolescents who embody strengths like cooperation and prudence often report a more robust sense of well-being in comparison to adults. In the context of the COVID-19 pandemic, Martínez-Martí’s survey of 348 Spanish subjects revealed that specific positive mental strengths, such as perseverance and intelligence, can notably enhance life satisfaction (Martínez-Martí et al., 2020). Additionally, research indicates that these positive mental traits can alleviate the impact of pandemic-related stress on depressive symptoms among adolescents (Liu and Wang, 2021).

After reviewing the relevant literature, it is evident that positive mental character is positively correlated with subjective well-being. However, direct evidence of a significant correlation between positive mental character and subjective well-being within the context of the COVID-19 pandemic is lacking in existing research. Consequently, this study aims to provide direct evidence of the relationship between the two during the COVID-19 pandemic. Based on the above, the research hypothesis of this paper is proposed:

*H2*: During the COVID-19 pandemic, the positive mental character of college students are significantly and positively associated with subjective well-being.

### 2.4 The relationship between exercise adherence and positive mental character

**Figure 1:**
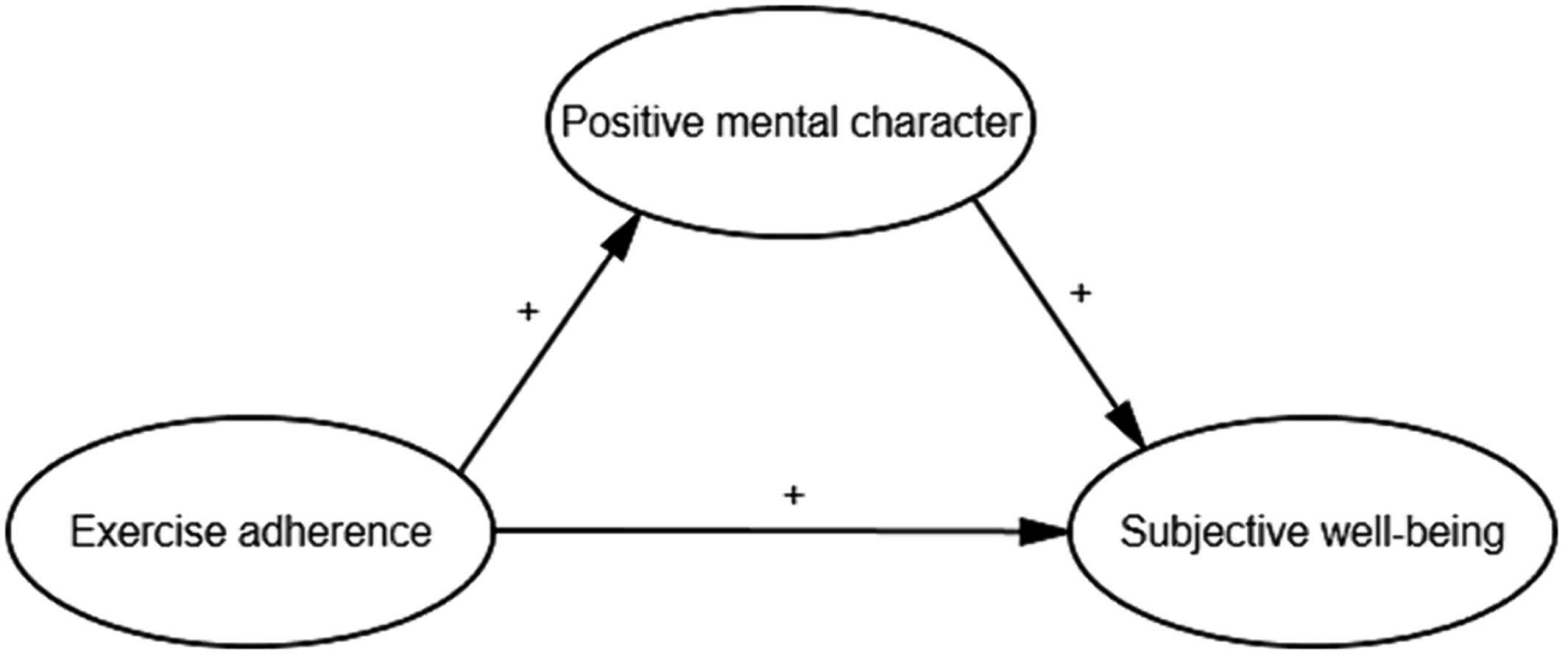
Hypothesized model of exercise adherence, positive mental character, and subjective well-being.

From an individual perspective, exercise adherence is understood as an internal psychological inclination to engage in physical activity on a long-term and regular basis (Sun et al., 2011), as well as an external behavioral tendency (Wang et al., 2018). The role of physical activity in fostering positive psychological experiences and mental health is well-documented and widely acknowledged across biological and psychological disciplines (Deslandes et al., 2009). Big data analysis reveals that the duration, frequency, and intensity of physical activity also show a correlation with positive mental character (Grasdalsmoen et al., 2020). The Personality-Environment Interaction Model posits that happiness is not solely determined by external environmental factors but is also shaped by an individual’s personality traits (Diener et al., 1999). This model suggests that happiness arises from the dynamic interplay between personality and environment. In light of this theory, the synergistic effect of physical activity and positive mental character is regarded as an effective approach to enhance subjective well-being. Consequently, exercise adherence can be posited as a precursor to the development of positive mental character. Prior studies have substantiated the mediating role of positive mental character when employed as a mediating variable, which underscores its potential significance in the current investigation. For instance, positive mental character have been shown to mediate, in part, the relationship between family support and subjective well-being (Lin, 2022). Similarly, these character exert a mediating influence when examining the impact of the school environment on the humanitarian care provided to college students (Xu et al., 2023). However, the psychological stress induced by the COVID-19 pandemic necessitates a re-evaluation of the role of positive mental character. In this study, exercise adherence is designated as the independent variable, positive mental character as the mediator, and subjective well-being as the dependent variable, aiming to investigate potential shifts in these relationships during the pandemic. The findings may offer valuable insights into the psychological state of college students during this period and inform strategies to enhance subjective well-being amidst the pandemic. In light of the foregoing, this study proposes the following research hypotheses and constructs the hypothesis model as shown in Figure 1.

*H3*: Exercise adherence was significantly and positively associated with positive mental qualities among college students during the COVID-19 pandemic.

*H4*: During the COVID-19 pandemic, college students’ exercise adherence was directly associated with subjective well-being and also indirectly associated with subjective well-being through positive psychological qualities.

## 3 Materials and methods

### 3.1 Samples and procedures

The study employed rigorous procedural controls during the design and distribution of the questionnaires. Initially, the reverse scoring items from the original scales were preserved in the questionnaire design to ensure consistency in measurement. Subsequently, a stratified sampling method was utilized to select a representative sample of seven colleges and universities in Henan Province, based on their academic standing and geographical distribution. The selected institutions included Zhengzhou University, Henan University, Henan University of Technology, Zhengzhou University of Aeronautics, Zhengzhou Normal University, Zhengzhou ShengDa University, and Henan Industry and Trade Vocational College. These universities were chosen to provide a comprehensive cross-section of the student population for the survey.

Given the constraints imposed by the COVID-19 pandemic, the survey was conducted using the online platform “Questionnaire Star,” which allowed for a secure and efficient data collection process. The questionnaire was disseminated from October 15, 2022, to December 14, 2022, with a target response rate that aimed to capture a significant portion of the student population’s perspectives during this unprecedented time. The initial page of the questionnaire included a statement of informed consent to ensure voluntary participation, accompanied by an information sheet detailing the survey’s purpose and emphasizing the anonymity of the responses and the authenticity of the participants’ answers. Two students from the target institutions were trained to assist in the distribution of the questionnaires, and they were instructed to provide a thorough explanation of the survey to ensure that each respondent fully comprehended the contents. The study protocol was approved by the relevant Human Research Ethics Committee, adhering to the ethical principles outlined in the Declaration of Helsinki. A total of 1,200 questionnaires were collected. During the screening process, 199 questionnaires were excluded due to patterns of highly similar responses or inconsistencies in the time taken to answer, which indicated potential inattentiveness or disengagement. This resulted in a final sample size of 1,001 for data analysis.

### 3.2 Research tool

#### 3.2.1 Exercise adherence scale

Exercise adherence among college students was assessed using the Exercise Adherence Scale (Wang et al., 2016). This scale comprises three dimensions: Behavioral Habits, Effort Commitment, and Emotional Experience, with a total of 14 items. A 5-point Likert scale was employed, where 1 indicates “not at all” and 5 indicates “completely.” Higher scores on the scale correspond to greater levels of exercise adherence. The internal consistency reliability of the questionnaire was determined using Cronbach’s alpha, which yielded coefficients of 0.893 for Behavioral Habits, 0.833 for Effort Commitment, and 0.830 for Emotional Experience, respectively, suggesting high internal consistency across the scale dimensions. The construct validity of the scale was further supported by the results of a confirmatory factor analysis, with the following fit indices: χ^2^/df = 2.896, CFI = 0.945, TLI = 0.932, NFI = 0.919, IFI = 0.945, GFI = 0.900, and RMSEA = 0.08, indicating an acceptable to good model fit.

#### 3.2.2 Positive mental character scale

Positive mental character was assessed using the Positive Mental Character Scale for College Students (Meng and Guan, 2009), which encompasses six dimensions: cognitive, affective, interpersonal, justice, moderation, and transcendence, comprising a total of 62 items. Responses were collected on a 5-point Likert scale, with higher scores indicating a more favorable profile of positive mental character. The reliability analysis yielded Cronbach’s alpha coefficients ranging from 0.829 to 0.849 for the six dimensions, demonstrating high internal consistency of the scale. The construct validity was further supported by the outcomes of the validation factor analysis, with fit indices indicating an excellent model fit: χ^2^/df = 1.733, CFI = 0.952, TLI = 0.927, NFI = 0.896, IFI = 0.953, GFI = 0.935, and RMSEA = 0.049.

#### 3.2.3 Subjective well-being scale

The Subjective Well-Being Scale employed in this study was a revised version of the International College Survey (Yan et al., 2011). This scale comprises three dimensions: life satisfaction, positive affect, and negative affect, with a total of 19 items. A 7-point Likert scale was utilized, where subjective well-being was determined by the aggregate of the life satisfaction, positive affect, and negative affect scores, with the negative affect score being reversely coded. Higher total scores on the scale signify a higher level of subjective well-being. The reliability analysis indicated good internal consistency, with Cronbach’s alpha coefficients of 0.806 for life satisfaction, 0.825 for positive affect, and 0.803 for negative affect, respectively. The validation factor analysis results further supported the scale’s construct validity, with fit indices suggesting an excellent model fit: χ^2^/df = 1.535, CFI = 0.999, TLI = 0.997, NFI = 0.912, IFI = 0.938, GFI = 0.909, and RMSEA = 0.029.

### 3.3 Data analysis

After preprocessing the collected data, SPSS 23.0 was utilized to assess for significant gender differences in exercise adherence, positive mental character, and subjective well-being using independent samples *T*-tests (*p* < 0.05 means significant). Mean, standard deviation and *t*-value were used as descriptive statistics. The relationships among the variables were examined using Pearson correlation tests. The Pearson correlation coefficient is used as a measure of correlation. The closer the Pearson coefficient is to 1, the higher the correlation. Hierarchical multiple regression analysis was conducted to ascertain the effects of the independent variable and the mediating role of positive mental traits on the dependent variable. Furthermore, structural equation modeling was performed using AMOS to validate the hypothesized mediating effects of positive mental traits.

## 4 Result

### 4.1 Common method biases

To assess potential method biases arising from common source, Harman’s single-factor test was employed. The analysis revealed that 11 factors with eigenvalues exceeding 1 were extracted, with the primary factor accounting for 29.929% of the variance. This percentage is below the 40% threshold, suggesting that the data in this study are not significantly affected by common method biases.

### 4.2 Difference analysis

Of the 1,001 samples in this study, there were 518 males and 483 females. Gender differences in exercise adherence, positive mental character, and subjective well-being among Chinese college students were examined using independent samples T-tests, with the results presented in Table 1. A significant difference was found in exercise adherence between genders (*p* < 0.001), with males exhibiting higher scores than females, indicating better exercise adherence for males during the COVID-19 pandemic. A similar pattern emerged for positive mental character, with a significant difference between genders (*p* < 0.01) and males reporting a stronger positive mental character than females. Additionally, a significant difference in subjective well-being was observed (*p* < 0.05), with male students’ scores being higher than those of females, suggesting that male students experienced a higher level of subjective well-being compared to their female counterparts during the pandemic.

**Table 1:** Analysis of differences in exercise adherence, positive mental character, and subjective well-being between genders.

Variables	Male (*N* = 518)	Female (483)	*t*
Exercise adherence scale	61.53 ± 12.94	50.77 ± 14.14	12.523***
Behavioral habits	23.28 ± 6.03	17.90 ± 6.01	14.122***
Effort commitment	18.21 ± 4.45	15.8 ± 4.95	8.082***
Emotional experience	20.04 ± 4.04	17.07 ± 5.03	10.244***
Positive mental characters scale	223.03 ± 37.04	216.91 ± 36.40	2.633**
Cognitive	42.93 ± 7.66	41.27 ± 7.60	3.443**
Interpersonal	36.69 ± 6.23	35.29 ± 6.17	3.584***
Affective	40.09 ± 7.30	39.65 ± 6.76	0.994
Moderation	35.54 ± 6.12	34.59 ± 6.36	2.422*
Justice	32.04 ± 5.80	31.46 ± 5.39	1.655
Transcendence	35.73 ± 6.48	34.67 ± 6.66	2.551*
Subjective well-being Scale	67.78 ± 14.59	65.68 ± 12.41	2.462*
Life satisfaction	21.52 ± 5.812	19.70 ± 5.385	5.147***
Positive affect	24.06 ± 8.018	24.23 ± 7.204	−0.358
Negative affect	22.20 ± 8.661	21.75 ± 7.47	0.906

*p < 0.05, **p < 0.01, ***p < 0.001.

### 4.3 Correlation analysis

Table 2 presents the outcomes of Pearson correlation analyses conducted to assess the presence of significant correlations among exercise adherence, positive mental character, and subjective well-being. The findings revealed a significant positive correlation (*p* < 0.01) among the three variables. Specifically, exercise adherence demonstrated the strongest correlation with subjective well-being, with a correlation coefficient of 0.511. Additionally, positive mental character also showed a significant positive correlation with both exercise adherence and subjective well-being, indicating a robust interrelationship among these constructs that underscores the importance of considering these factors in conjunction when examining overall well-being.

**Table 2:** Correlation analysis of exercise adherence, positive mental character, and subjective well-being.

	Exercise adherence	Positive mental character	Subjective well-being
Exercise adherence	1		
Positive mental character	0.511**	1	
Subjective well-being	0.341**	0.472**	1

*p < 0.05, **p < 0.01, ***p < 0.001.

### 4.4 Regression analysis

The mediation analysis was conducted following the procedure suggested by Baron and Kenny (1986). Initially, the significance of the “c” path was assessed by regressing the dependent variable on the independent variable, controlling for the effects of any control variables. Subsequently, the significance of the “a” path was determined by regressing the independent variable on the mediator variable. Finally, the mediating variable was used to regress the dependent variable, while controlling for the independent variables, to test the significance of coefficients “b” and “c’”. Table 3 displays the outcomes of the regression analyses. Model 1, which includes only control variables, revealed their effect on positive mental character. In Model 2, after accounting for control variables, exercise adherence was found to significantly and positively influence positive mental character (*β* = 0.54, *p* < 0.001), thus supporting Hypothesis 3. Model 3, also with control variables only, showed their impact on subjective well-being. Model 4 indicated that exercise adherence significantly and positively affected subjective well-being (*β* = 0.35, *p* < 0.001), confirming Hypothesis 1. Model 5 demonstrated that positive mental character significantly and positively affected subjective well-being when controlling for the independent variables (*β* = 0.40, *p* < 0.001), verifying Hypothesis 2.

**Table 3:** Regression analysis of exercise adherence, positive mental character, and subjective well-being.

Variables	Positive mental character		Subjective well-being		
	Model 1	Model 2	Model 3	Model 4	Model 5
Constant	216.94	123.15	67.02	44.45	26.29
Genders	−0.08	0.12	−0.08	0.05	0.006
Exercise adherence		0.54***		0.35***	0.14***
Positive mental character					0.40***
*R*^2^	0.04	0.28	0.02	0.12	0.24
Δ*R*^2^	0.04	0.25	0.02	0.11	0.11
F	18.32***	131.17***	8.89***	46.40***	77.21***
ΔF	18.32***	344.28***	8.89***	119.31***	149.00***

*p < 0.05, **p < 0.01, ***p < 0.001. The coefficients in the table are standardized regression coefficients.

### 4.5 Indirect effect test

**Figure 2:**
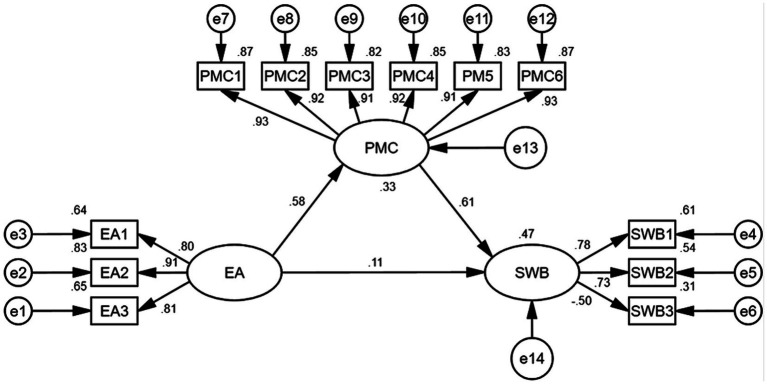
Structural equation model of exercise adherence, positive mental character, and subjective well-being. EA, exercise adherence; EA1, behavioral habits; EA2, effort commitment; EA3, emotional experiences; PMC, positive mental character; PMC1, cognitive; PMC2, interpersonal; PMC3, affective; PMC4, moderation; PMC5, justice; PMC6, transcendence; SWB, subjective well-being; SWB1, life satisfaction; SWB2, positive affect; SWB3, negative affect.

Figure 2 illustrates the structural equation modeling depicting the relationships among exercise adherence, positive mental character, and subjective well-being. The model fit indices indicated a good fit: χ^2^/df = 1.926; GFI = 0.953; AGFI = 0.926; CFI = 0.978; and RMSEA = 0.070. To validate the mediating role of positive mental character, Bootstrap resampling was employed, with the sample size “k” set to 5,000 and a 95% confidence interval. As presented in Table 4, both the direct and indirect effects were found to be statistically significant, as the 95% confidence intervals did not encompass “0.” This suggests that the exercise adherence of Chinese college students has a significant positive impact on subjective well-being during the COVID-19 pandemic, both directly and indirectly through positive mental character, thus confirming Hypothesis 4.

**Table 4:** Bootstrap test for the mediating role of positive mental character.

	Effects	Estimate	95% confidence interval	
Exercise adherence → Positive mental character → Subjective well-being	Direct effects	0.11	0.029	0.191
	Indirect effects	0.35	0.303	0.406

## 5 Discussion

The mean for exercise adherence scale among Chinese college students was 56.33, slightly exceeding the median of 56.00. For positive mental character scale, the mean stood at 220.08, marginally higher than the median of 219.00. The mean for subjective well-being scale was 67.80, which was slightly below the median of 68. Comparatively, a pre-pandemic study reported a higher mean for subjective well-being among college students, at 73.94 (Yang, 2020). This suggests that the subjective well-being of college students has been notably impacted by the COVID-19 pandemic. Consistent with this finding, studies from other regions have also indicated that the perceived risk of the COVID-19 pandemic significantly diminishes individuals’ sense of well-being (Yıldırım and Güler, 2021). Because the extent of well-being reduction was found to correlate with the perceived risk of infection and the potential harm the virus could inflict on one’s health and life (Yang and Ma, 2020).

Regarding gender differences, the data indicated that across all measured dimensions—exercise adherence, positive mental character, and subjective well-being—there was a significant disparity, with males consistently scoring higher than females. This pattern aligns with findings from previous research (Huang, 2021). The observed gender difference in exercise adherence might stem from the fact that boys tend to have greater intrinsic motivation to engage in sports activities compared to girls (Fuentealba-Urra et al., 2022). The significant differences in positive mental traits between genders may be linked to certain personality characteristics. Males, who tend to score higher on openness and extraversion, may be more adept at managing negative emotions during adverse events (Zhang et al., 2024). This propensity could contribute to a more robust positive mental character among males. Additionally, given the evidence that physical activity can bolster positive psychological qualities, the higher levels of sports participation observed in males may further enhance their positive mental character (Wang et al., 2024). The disparities in subjective well-being could stem from multiple factors. Theories such as the Fulfillment and Engagement theories posit that the satisfaction of needs and the attainment of goals are instrumental in fostering happiness, while unmet needs and unachieved goals can lead to unhappiness (Das et al., 2020). Anxiety is a critical factor influencing subjective well-being, and research has shown that females are generally more prone to experiencing anxiety (McHenry et al., 2014). This heightened susceptibility to anxiety can exacerbate stress and negatively impact mental health. Furthermore, studies suggest that women often perceive the virus as posing a greater risk to their health, which may lead to increased worry and more negative mental states (Metin et al., 2022). In addition, the lower levels of exercise adherence among females could also play a role in their reduced subjective well-being. Yuan and You (2022) also found that the physical activity during the epidemic was significantly related to the subjective well-being. Given the potential benefits of physical activity for mental health and well-being, the lower engagement in regular exercise among females may contribute to less optimal psychological outcomes during the COVID-19 pandemic.

In the correlation analysis, a significant positive correlation was identified among exercise adherence, positive mental character, and subjective well-being among Chinese college students during the COVID-19 pandemic, as determined using Pearson’s correlation coefficient. While previous studies have established correlations between these variables (Williams, 2008; Vela et al., 2016; Brand et al., 2020), the confirmation of these relationships within the specific context of the pandemic was lacking. The present study not only affirms the existence of significant positive correlations between the three variables during the pandemic but also highlights the importance of these relationships for understanding and potentially mitigating the mental health impacts of such crises. As other researchers have demonstrated, residents who are inactive and physically inactive are more likely to have anxiety, depression, sleep disorders and lower subjective well-being (Li et al., 2022). The study’s findings revealed that exercise adherence demonstrated the highest significant correlation with positive mental character (0.511), indicating a strong positive relationship. And the benefits of physical activity in promoting the emergence of a positive mental have been widely recognized by scholars (Giandonato et al., 2021). This was followed by the correlation between positive mental character and subjective well-being (0.472), and the lowest correlation was observed between exercise adherence and subjective well-being (0.341), both of which were also significant. According to Norrish and Vella-Brodrick (2009), positive mental enhances people’s satisfaction and feelings of happiness in life, thus contributing to an individual’s subjective well-being. Physical activity and positive psychology also help to eliminate negative emotions and increase the level of subjective well-being when people face stress and anxiety from potential threats (Wolf et al., 2021; Zhao et al., 2023). Tests of mediation effects suggested that positive mental characters exert a partial mediating effect on the relationship between exercise adherence and subjective well-being. A study of 826 college students also showed that positive psychology can indirectly affect college students’ subjective well-being (Wang et al., 2022). Other researchers’ studies have shown that positive mental can indirectly affect college students’ emotion regulation ability (Mu et al., 2024). It also provides evidence that college students experience higher levels of subjective well-being. This study further demonstrates that it is feasible to enhance college students’ perceptions of well-being during the COVID-19 pandemic by promoting persistent exercise to bolster their positive mental character.

This study demonstrated a significant positive association between exercise adherence, positive mental character, and subjective well-being among college students in the context of the COVID-19 pandemic. The findings of this study carry significant implications for enhancing positive mental characters and improving subjective well-being in the face of future epidemics or large-scale health emergencies. That is, increasing exercise adherence among college students will contribute to the development of positive mental character and subjective well-being, even in the face of a global public health event. Therefore, developing an awareness of exercise and a lifelong exercise habit among college students is an important part of the formation of a positive mental among college students, as well as experiencing a higher level of subjective well-being in the context of the global pandemic. Secondly, attention should also be paid to the development of positive mental character, which are equally effective in eliminating negative emotions and experiencing high levels of subjective well-being among college students. Finally, the results of this study also enrich the findings of exercise psychology and positive psychology. This study demonstrates for the first time that exercise adherence, positive mental character, and subjective well-being are positively and significantly associated even in the context of the COVID-19 pandemic.

## 6 Conclusion

The results indicated significant gender differences in exercise adherence, positive mental character, and subjective well-being, with males exhibiting higher mean scores than females. Throughout the COVID-19 pandemic, exercise adherence continued to have a significantly positively associated with positive mental character and subjective well-being. Additionally, positive mental character was found to significantly and positively associated with subjective well-being. The association between exercise adherence and subjective well-being is both direct and indirect, mediated by positive mental character.

### 6.1 Limitations and future directions

Firstly, this study’s participants were limited to college students who had not yet graduated at the time of the survey, excluding other potential groups such as those who had graduated or were not enrolled at the time. Future research could expand to include these additional groups to provide a more comprehensive understanding of the impact of the COVID-19 pandemic on various student populations.

Secondly, the psychological states of college students during the COVID-19 pandemic might be distinct from those experienced during other pandemics. Comparing the psychological states of college students across different pandemics could be a valuable direction for future research, shedding light on the specific impacts and the resilience of this demographic in the face of widespread health crises.

Thirdly, subjective well-being is associated by multiple factors, with the perception of the virus’s potential threat being one of the key influences during the COVID-19 pandemic. This study focused on the association between exercise adherence, positive mental character, and subjective well-being. Future studies could broaden the scope to include a wider array of factors, such as social support, mental health services, and academic pressures, to provide a more nuanced understanding of the determinants of well-being.

Lastly, while this study established the relationship between exercise adherence, positive mental character, and subjective well-being during the COVID-19 pandemic, it did not employ qualitative methods such as interviews to delve deeper into the psychological conditions of students. Future research could benefit from employing grounded theory, case studies, and other qualitative approaches to gain richer, more nuanced insights into the psychological condition of college students during pandemic times.

## Data availability statement

The original contributions presented in the study are included in the article/supplementary material, further inquiries can be directed to the corresponding authors.

## Ethics statement

The studies involving humans were approved by Ethics Committee of Zhengzhou University. The studies were conducted in accordance with the local legislation and institutional requirements. Written informed consent for participation in this study was provided by the participants’ legal guardians/next of kin.

## Author contributions

YC: Investigation, Methodology, Writing – original draft, Formal analysis. NF: Formal analysis, Investigation, Validation, Writing – original draft. YZ: Data curation, Investigation, Resources, Writing – review & editing. ZL: Data curation, Investigation, Supervision, Writing – review & editing. QZ: Conceptualization, Project administration, Resources, Supervision, Visualization, Writing – review & editing.
